# Thin-Layer Drying Kinetics and Quality Attributes of Apple Pomace Powders from Different Varieties

**DOI:** 10.3390/foods15061090

**Published:** 2026-03-20

**Authors:** Liliana Ceclu, Alexandru Radu Corbu, Iurie Rumeus, Alexandra Cicanci, Ana-Maria Blejan, Irina Vîșcu, Violeta Nour

**Affiliations:** 1 Faculty of Economics, Engineering and Applied Sciences, Cahul State University “B.P. Hasdeu”, Piaţa Independenţei, 1, 3900 Cahul, Moldova; liliana.ceclu@feisa.usch.md (L.C.); iurie.rumeus@feisa.usch.md (I.R.); alexandra.cicanci@feisa.usch.md (A.C.); irina.viscu@feisa.usch.md (I.V.); 2 Department of Horticulture & Food Science, University of Craiova, 13 AI Cuza Street, 200585 Craiova, Romania; corbu_lx@yahoo.co.uk; 3 Faculty of Agronomy, University of Craiova, Libertății Street, 19, 200583 Craiova, Romania; anamariablejan11@gmail.com

**Keywords:** apple pomace, drying kinetics, mathematical models, apple variety, phenolic compounds, antioxidant activity

## Abstract

Apple pomace, a major by-product of juice processing, requires effective drying for valorization and preservation. This study investigated the thin-layer drying kinetics of apple pomace from three varieties (Starkrimson, Idared, and Jonagold) under forced convection at 57 °C, 63 °C, and 68 °C. Seven mathematical models were used to fit the experimental moisture ratio data and evaluated using statistical indicators (*R*^2^, *RMSE*, *χ*^2^, and *MBE*). In addition, the proximate composition, titratable acidity, water activity, color parameters, rehydration capacity, total phenolic content, antioxidant activity, and phenolic and organic acid profiles of the resulting apple pomace powders were assessed. Of the models tested, the Midilli–Kucuk model demonstrated superior performance with the highest *R*^2^ (>0.99) and lowest *RMSE* and *χ*^2^ values across all varieties and temperatures. The Page and Logarithmic models also showed good predictive capability. Significant differences in phenolic content and antioxidant activity were observed between powders from different apple varieties, with Starkrimson showing the highest values, followed by Jonagold and Idared. These findings provide essential kinetic parameters for optimizing apple pomace drying processes and support the development of value-added products from this abundant agro-industrial by-product.

## 1. Introduction

Apple (*Malus domestica*) ranks among the most widely cultivated and consumed fruit crops globally, with world production exceeding 95 million tons annually [1]. The juice processing industry generates substantial quantities of apple pomace, a solid residue comprising peel, core, seeds, and pulp, representing approximately 25–30% of the original fruit mass [2,3]. This by-product, with its high moisture content (75–85% wet basis), is prone to rapid microbial deterioration and poses significant disposal challenges for processors [4,5,6,7].

Apple pomace is rich in polyphenols, dietary fiber, minerals, and bioactive compounds, which makes it a valuable resource for valorization into functional food ingredients, animal feed supplements, and bioenergy feedstocks [2,7]. However, its high moisture content necessitates immediate stabilization to prevent spoilage and enable storage, transportation, and further processing. Drying is the most widely employed preservation method, lowering water activity to suppress microbial growth while concentrating nutrients and extending shelf life. A final moisture content of maximum 10% is recommended to maintain the stability and shelf life of fruit pomace during storage, with some studies recommending 6–8% for maximum stability [8,9].

Thin-layer drying under forced convection is a common industrial approach for processing fruit pomaces, offering controlled mass and heat transfer conditions. Understanding the drying kinetics is essential for optimizing process parameters, minimizing energy consumption, and maintaining product quality [10,11,12]. Mathematical modeling of drying kinetics enables accurate prediction of moisture removal rates, estimation of drying times, and calculation of transport properties such as activation energy and effective moisture diffusivity [13,14,15,16].

Various empirical and semi-empirical models have been developed to describe moisture removal during the drying process, each differing in structural complexity and physical interpretation [17,18,19,20]. The selection of an appropriate model depends on the characteristics of the material, the drying conditions, and the balance between predictive accuracy and practical applicability [17,20,21]. In the case of apple pomace, which exhibits complex moisture transport behavior due to its heterogeneous structure and high initial moisture content, a comparative evaluation of multiple models across different varieties is necessary to identify reliable predictive tools suitable for industrial implementation.

Previous studies have investigated the drying kinetics of apple pomace and reported varying model preferences. Kara and Doymaz [2] found the Midilli model best described apple pomace drying at 50–70 °C, with effective diffusivity values of 1.73–4.40 × 10^−10^ m^2^/s and activation energy of 29.65 kJ/mol. Araujo et al. [4] reported that the Crank diffusion model fitted well hot-air drying of apple–ginger mixed pomace, with diffusivity ranging from 2.28 to 4.83 × 10^−10^ m^2^/s and activation energy of 23.9 kJ/mol. However, comprehensive comparative assessments of multiple mathematical models specifically for apple pomace across different varieties remain limited in the scientific literature [22].

The influence of apple variety on drying kinetics has received limited attention, despite known differences in cellular structure, pectin content, and initial moisture distribution among cultivars [9,23]. Furthermore, systematic comparison of a broad spectrum of mathematical models using multiple statistical indicators can provide robust model selection and improve predictive accuracy for industrial applications [18,24,25,26].

Therefore, this study aims to compare the thin-layer drying kinetics of pomace obtained from three commercially important apple varieties (Starkrimson, Idared, and Jonagold) under identical forced convection conditions. Several mathematical models are evaluated to identify the most accurate predictive tools for each variety. Additionally, variety-specific values of effective moisture diffusivity and activation energy are determined. The physicochemical properties, bioactive compounds, and antioxidant activity of the resulting powders are also comprehensively characterized in order to establish relationships between apple variety, drying conditions, and final product quality. This integrated approach provides both fundamental kinetic parameters and practical quality benchmarks that are essential for optimizing industrial strategies for apple pomace valorization.

## 2. Materials and Methods

### 2.1. Sample Preparation

Apple pomace was obtained from juice extraction of three commercial apple varieties: Starkrimson (red cultivar), Idared (red-striped cultivar), and Jonagold (yellow-red bicolor cultivar). These varieties were selected based on their widespread cultivation and commercial importance in juice processing operations. Apples were harvested at commercial maturity from commercial orchards in Dolj County (Romania) during the 2025 harvest season. Fruits were stored under controlled conditions (2–4 °C, 90–95% relative humidity) for 2 weeks before processing to minimize compositional changes. Apple pomace was obtained under laboratory conditions using a centrifugal juice extractor (R.G.V., Como, Italy). Fresh pomace samples were collected immediately after juice extraction and promptly prepared for the drying process.

The pomace, consisting of peel, core, seeds, and pulp fragments, was ground using a laboratory blender (Tefal Smart, MB450141, Istanbul, Turkey) for 2 min to achieve uniform particle size and consistency, facilitating even spreading and reproducible drying conditions. Initial moisture content was assessed using the gravimetric method, where samples were dried at 105 °C until a constant weight was reached, according to AOAC guidelines (AOAC 925.10) [27].

### 2.2. Drying Process

Thin-layer drying experiments were conducted using a laboratory-scale forced convection hot air dryer (Deca + SS Design, Profimatic, Cluj-Napoca, Romania) equipped with temperature control (±1 °C) and air circulation system. Drying was performed at three preset temperatures: 57 °C, 63 °C, and 68 °C. These temperatures were selected to balance drying efficiency with quality preservation, as excessive temperatures can degrade heat-sensitive bioactive compounds.

For each experiment, approximately 120–125 g of homogenized pomace was spread uniformly in a thin layer (2 mm thickness) on parchment paper covering a circular surface with 26 cm diameter. The thin-layer configuration ensures negligible internal temperature gradients and allows the drying process to be controlled primarily by external heat and mass transfer conditions. Samples were placed on perforated trays to allow air circulation from both top and bottom surfaces. Hot air was circulated using a fan at a constant velocity of approximately 1.5 m/s, as specified in the equipment manual, to ensure uniform heat and mass transfer across the sample surface.

Sample mass was measured at 30 min intervals using a digital balance with 0.001 g precision until the moisture content reached equilibrium (constant weight). Each drying experiment was carried out in triplicate to ensure reproducibility and statistical validity. Drying was continued until the samples reached approximately 7–8% moisture content (wet basis), suitable for stable storage.

The dried materials were ground using a household electric grinder, passed through a 0.5 mm sieve, packed in bags and stored at 20 °C for subsequent analyses.

Figure S1 (available in the “Supplementary Materials”) presents the appearance of the apple pomace uniformly spread in a thin layer (2 mm thickness) on parchment paper covering a circular surface with 26 cm diameter (1) and of the apple pomace powder (2) for Starkrimson (a), Idared (b) and Jonagold (c) varieties.

### 2.3. Drying Kinetics

The drying kinetics of apple pomace were characterized using dimensionless moisture ratio (*MR*) and drying rate (*DR*) to describe the temporal evolution of moisture content during thin-layer forced convection drying [11].

The moisture ratio (*MR*), a parameter representing the relative moisture content at any time during drying, was evaluated using Equation (1):

(1) $$MR=\frac{M_{t}-M_{e}}{M_{0}-M_{e}}$$

where *M_t_* is the moisture content at time *t* (kg water/kg dry matter), *M*_0_ is the initial moisture content (kg water/kg dry matter), and *Me* is the equilibrium moisture content (kg water/kg dry matter).

For practical purposes, since equilibrium moisture content is negligible compared to initial and instantaneous moisture contents, the moisture ratio can be simplified to Equation (2) [18,19,24]:

(2) $$MR=\frac{M_{t}}{M_{0}}$$

This simplified form was used in the present study for model fitting, consistent with common practice in thin-layer drying research [5,6,24].

The drying rate (*DR*) was calculated from the slope of the moisture content versus time curve using Equation (3) and expressed as kg water/(kg dry matter·min).

(3) $$DR=\frac{M_{t+\Delta t}-M_{t}}{\Delta t}$$

where *M_t_*_+Δ*t*_ and *M_t_* are the moisture contents at times *t* + Δ*t* and *t*, respectively, and Δ*t* is the time interval (30 min).

Effective moisture diffusivity (*D_eff_*) characterizes the rate of internal moisture migration during drying and is of interest for dryer design and process optimization [2]. For thin-layer drying of slab geometry, Fick’s second law of diffusion provides the theoretical basis for calculating *D_eff_*. The analytical solution for infinite slab geometry with uniform initial moisture distribution and negligible external resistance is given by Equation (4) [5,6,28]:

(4) $$MR=\frac{8}{\pi^{2}}\sum_{n=0}^{\infty }\frac{1}{(2n+1{)}^{2}}\exp\left[-\frac{(2n+1{)}^{2}\pi^{2}D_{eff}t}{4L^{2}}\right]$$

where *L* is the half-thickness of the slab (m), *t* is drying time (s), and *n* is a positive integer.

For long drying times (*MR* < 0.6), the series can be truncated to the first term with negligible error, yielding Equation (5) [18,29]:

(5) $$MR=\frac{8}{\pi^{2}}\exp\left[-\frac{\pi^{2}D_{eff}t}{4L^{2}}\right]$$

By applying the natural logarithm to both sides, a linear relationship is obtained (Equation (6)):

(6) $$\ln\left(MR\right)=\ln\left(\frac{8}{\pi^{2}}\right)-\frac{\pi^{2}D_{eff}}{4L^{2}}t$$

The effective moisture diffusivity was determined from the slope (*k*) of the linear plot of ln(*MR*) against time according to Equation (7):

(7) $$D_{eff}=-\frac{4L^{2}k}{\pi^{2}}$$

where the slope *k* = π^2^*D_eff_*/(4*L*^2^). For the present study, the half-thickness *L* was 0.001 m (1 mm for a 2 mm layer) [5].

Effective moisture diffusivity exhibits an Arrhenius-type dependence on temperature (Equation (8)) [2,5,18,28]:

(8) $$D_{eff}=D_{0}\exp\left(-\frac{E_{a}}{RT}\right)$$

where *D*_0_ is the pre-exponential factor (m^2^/s), *Ea* is the activation energy (kJ/mol), *R* is the universal gas constant (8.314 J/(mol·K)), and *T* is the absolute temperature (K).

Taking the natural logarithm of Equation (8) yields a linear form (Equation (9)):

(9) $$\ln\left(D_{eff}\right)=\ln\left(D_{0}\right)-\frac{E_{a}}{R}\cdot \frac{1}{T}$$

The activation energy was obtained from the slope of ln(*D_eff_*) versus 1/*T*, as described by Equation (10):

(10) $$E_{a}=-R\times \mathrm{slope}$$

The activation energy corresponds to the minimum energy necessary to trigger moisture diffusion and provides insight into the temperature sensitivity of the drying process [28].

### 2.4. Mathematical Modeling and Statistical Analysis

The experimental moisture ratio data were fitted to seven widely used thin-layer drying models to describe the drying kinetics of apple pomace. These models include empirical, semi-empirical, and theoretical formulations commonly applied in food drying research [14,18,24,25,30,31,32]. The models tested are presented in Table 1.

The fit quality of each mathematical model was assessed using four statistical parameters: coefficient of determination (*R*^2^), root mean square error (*RMSE*), reduced chi-square (*χ*^2^), and mean bias error (*MBE*) [25,30,33]. These criteria are calculated using Equations (11)–(14):

Coefficient of determination (*R*^2^):

(11) $$R^{2}=1-\frac{\sum_{i=1}^{N}(MR_{exp,i}-MR_{pre,i}{)}^{2}}{\sum_{i=1}^{N}(MR_{exp,i}-{\bar{MR}}_{exp}{)}^{2}}$$

Root mean square error (*RMSE*):

(12) $$RMSE={\left[\frac{1}{N}\sum_{i=1}^{N}(MR_{pre,i}-MR_{exp,i}{)}^{2}\right]}^{1/2}$$

Reduced chi-square (*χ*^2^):

(13) $$\chi^{2}=\frac{\sum_{i=1}^{N}(MR_{exp,i}-MR_{pre,i}{)}^{2}}{N-n}$$

Mean bias error (*MBE*):

(14) $$MBE=\frac{1}{N}\sum_{i=1}^{N}(MR_{pre,i}-MR_{exp,i})$$

where *MR_exp_*_,_*_i_* is the *i*-th experimentally observed moisture ratio, *MR_pre_*_,_*_i_* is the *i*-th predicted moisture ratio, ${\bar{MR}}_{exp}$ is the mean experimental moisture ratio, *N* is the number of observations, and *n* is the number of model parameters.

The best model was selected based on the highest *R*^2^ value and the lowest *RMSE*, *χ*^2^, and *MBE* values [2,30]. A good model fit is indicated by *R*^2^ approaching 1.0, and *RMSE*, *χ*^2^, and *MBE* approaching zero [5,6,33].

Mathematical modeling and curve fitting analyses were performed using CurveExpert Professional software (version 2.6.5, Hyams Development, Madison, AL, USA), a cross-platform tool designed for curve fitting and data analysis. Data can be modelled using a toolbox of linear regression models, nonlinear regression models, smoothing methods, or various kinds of splines. Non-linear regression analysis was performed using the Levenberg–Marquardt algorithm.

### 2.5. Color

A PCE-CSM1 colorimeter (PCE Instruments, Southampton, UK), calibrated with a white standard, was used to measure the color of the dried pomace powders. For each sample, three replicates were analyzed, and six measurements per replicate were taken to record the color coordinates L* (lightness), a* (redness/greenness), and b* (yellowness/blueness). Chroma (C*) and hue angle (h*) were determined as (a*^2^ + b*^2^)^1/2^ and arctan(b*/a*), respectively.

### 2.6. Water Activity

To determine water activity (a_w_), apple pomace powders were analyzed using a Humimeter RH2 water activity meter (Schaller Messtechnik GmbH, Max-Schaller-Straße, Styria, Austria).

### 2.7. Rehydration Ratio

Rehydration ratio was determined according to Waseem et al. [35]. Briefly, 2.5 g of apple pomace powder were soaked for 5 min in 50 mL of boiling water then filtered through Whatman filter paper 1. The rehydrated sample was weighed using an electronic balance, and the rehydration ratio was determined as the weight of the rehydrated sample divided by that of the dried sample.

### 2.8. Proximate Composition

Dry matter (984.25), crude protein (950.36), crude fat (935.38), dietary fiber (985.29) and ash (925.51) content of apple pomace powders was assessed following standard AOAC procedures [27].

### 2.9. Titratable Acidity

An aqueous extract was prepared by homogenizing 5 g of apple pomace powder and diluting it to 100 mL with distilled water. The slurry obtained was passed through Whatman No. 1 filter paper to eliminate solid residues. A 10 mL aliquot of the filtrate was titrated to pH 8.2 with 0.1 N NaOH, and the titration results were expressed assuming malic acid as the main acid in apple pomace.

### 2.10. Phenolic Compound Extraction

Phenolic compounds were extracted from dried pomace samples by sonication in methanol for 60 min at room temperature. After centrifugation at 6000 rpm during 5 min, the supernatants were filtered through 0.45 μm membranes. The filtrates were further evaluated for total phenolic content, antioxidant activity using the DPPH assay, and phenolic compound profiling by RP-HPLC.

### 2.11. Total Phenolic Content

The methanolic extracts were analysed for total phenolic content using the Folin–Ciocalteu spectrophotometric method according to Singleton et al. [36]. Results were expressed as mg gallic acid equivalents (GAE) per 100 g dry pomace, based on a gallic acid calibration curve.

### 2.12. DPPH Radical Scavenging Activity

The DPPH radical scavenging activity of pomace powders was assessed according to the spectrophotometric method described by Brand-Williams et al. [37]. Trolox was used as a standard, and results were reported as mmol Trolox per 100 g of sample.

### 2.13. Phenolic Profile

Individual phenolic compounds were quantified in the methanolic extracts of apple pomace powders by HPLC according to Nour et al. [38], using a Finnigan Surveyor Plus system (Thermo Electron Corporation, San Jose, CA, USA) fitted with a diode-array detector. Phenolic compound concentrations were reported as mg per 100 g of apple pomace powder.

### 2.14. Organic Acid Profile

High-performance liquid chromatography (HPLC) was used to detect and quantify organic acids in the aqueous extracts of apple pomace powders, according to the procedure of Nour et al. [39]. Organic acid concentrations were reported as mg per 100 g of apple pomace powder.

### 2.15. Statistical Analysis

Statistical analyses of the experimental data were carried out using Statgraphics Centurion XVI (StatPoint Technologies, Warrenton, VA, USA). All assays were conducted at least in triplicate, and results are expressed as mean ± standard deviation. ANOVA (*p* < 0.05) followed by the LSD test was used for multiple comparisons.

## 3. Results and Discussion

### 3.1. Drying Curves

**Figure 1:**
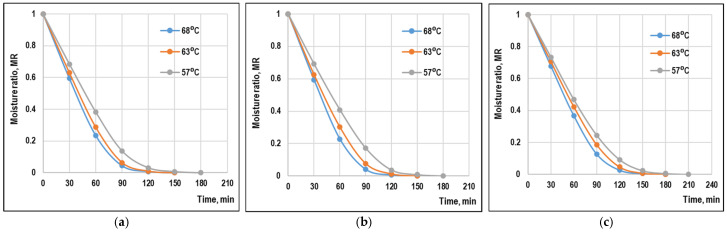
Variation in moisture ratio of apple pomace samples as a function of drying air temperature and drying time: (**a**) Idared, (**b**) Jonagold, (**c**) Starkrimson.

The variation in moisture ratio with drying time for apple pomace from three varieties (Starkrimson, Idared, and Jonagold) at 57 °C, 63 °C, and 68 °C is shown in Figure 1. The drying curves exhibited typical falling-rate behavior with no constant-rate period, indicating that internal moisture diffusion was the predominant mechanism controlling the drying process [2,5,6]. This behavior is characteristic for materials that are highly hygroscopic, possess high initial moisture content, and exhibit a porous cellular structure [28,31].

### 3.2. Drying Rate

**Figure 2:**
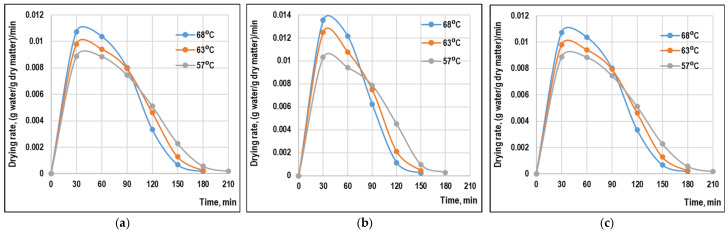
Variation in the drying rate of apple pomace samples during the drying process: (**a**) Idared, (**b**) Jonagold, (**c**) Starkrimson.

Figure 2 presents the drying rate curves as a function of drying time at different drying temperatures for the three apple varieties. All curves exhibited continuously decreasing drying rates throughout the process, confirming that the process lacked a constant-rate period and indicating that drying took place entirely in the falling-rate period [2,5,6]. This behavior is typical of biological materials where internal moisture diffusion controls the drying process [28].

Initial drying rates were highest at elevated temperatures, with maximum values observed at 68 °C for all varieties. For Starkrimson, the initial drying rate increased from 0.00886 kg water/(kg dry matter·min) at 57 °C to 0.01073 kg water/(kg dry matter·min) at 68 °C. Similar trends were noted for Idared and Jonagold varieties, with initial drying rates ranging from 0.01030 to 0.01354 kg water/(kg dry matter·min) across the temperature range studied. The drying rate exhibited a sharp decrease during the first 60–90 min, then declined more gradually as moisture content approached equilibrium. This pattern reflects the progressive reduction in moisture content, decreased moisture diffusivity at lower moisture levels, and increased resistance to moisture transport as the product structure shrinks and pores collapse. The rapid initial decrease in drying rate is associated with removal of surface and loosely bound water, while the slower decline at later stages corresponds to removal of more tightly bound water requiring greater energy for desorption [33].

Temperature exerted a pronounced influence on drying rate throughout the process. Higher temperatures maintained elevated drying rates for longer periods, resulting in faster overall drying. This effect is attributed to increased vapor pressure at the product surface, enhanced moisture diffusivity, and reduced air relative humidity at higher temperatures [31,33].

### 3.3. Effective Diffusivity

Table 2 presents the effective moisture diffusivity values calculated from the slopes of ln(*MR*) versus time plots. The diffusivity values ranged from 1.167 × 10^−8^ to 1.698 × 10^−8^ m^2^/s across all varieties and temperatures, falling within the typical range previously reported for food materials (10^−11^ to 10^−9^ m^2^/s) [2,5,26,40].

A clear positive correlation between drying temperature and effective moisture diffusivity was observed for all three apple varieties. All three apple varieties demonstrated a clear positive correlation between temperature and effective diffusivity. As drying temperature increased from 57 °C to 68 °C, D*_eff_* values exhibited a consistent upward trend. At each temperature level, the three apple varieties exhibited different effective diffusivity values, reflecting differences in their cellular structure, composition, and porosity. Notably, Jonagold showed the highest diffusivity at the highest temperature, suggesting greater temperature sensitivity.

*R*^2^ values ranging from 0.9186 to 0.9556 confirmed the strong fit of the diffusion model to the experimental observations. All values exceeded 0.91, demonstrating that Fick’s diffusion law adequately described the moisture removal process in apple pomace across all varieties and temperatures tested. At elevated temperatures, rapid surface drying can create a dense outer layer that impedes internal moisture migration, effectively reducing the apparent diffusivity despite higher thermal energy. Additionally, the simplified Fick’s law model assumes constant diffusivity and negligible shrinkage, which may not fully capture the complex moisture transport phenomena in pomace materials undergoing significant structural changes [15,29,41].

Among varieties, Jonagold exhibited the highest diffusivity at 57 °C (1.698 × 10^−8^ m^2^/s), followed by Idared (1.641 × 10^−8^ m^2^/s) and Starkrimson (1.423 × 10^−8^ m^2^/s). These differences may reflect variations in cellular structure, porosity, and pectin content among cultivars. The Fickian diffusion model provided a good fit to the experimental data, with *R*^2^ values for ln(*MR*) versus time regression ranging from 0.9186 to 0.9556 [2].

Comparison with literature values reveals that the diffusivity values obtained in this study are approximately two orders of magnitude higher than those reported by Kara and Doymaz (1.73–4.40 × 10^−10^ m^2^/s) for apple pomace dried at 50–70 °C [2], and by Araujo et al. [4] (2.28–4.83 × 10^−10^ m^2^/s) for apple–ginger mixed pomace, but closer to those determined by Wang et al. [5] (1.9082–3.9346 × 10^−9^ m^2^/s) for hot air drying of thin-layer apple pomace, Wang et al. [6] (1.0465 × 10^−8^ m^2^/s) for microwave drying of apple pomace, and by Shekarau et al. [28] (9.02015 × 10^−9^–1.53000 × 10^−8^) for cashew apple pomace. The observed discrepancy could be attributed to variations in sample preparation (particle size, layer thickness), drying equipment, and calculation methodology. The present study used a relatively thin layer (2 mm) and ground pomace, which may facilitate faster moisture migration compared to thicker layers or coarser particles [4,28].

### 3.4. Activation Energy

The activation energy for moisture diffusion was determined from the Arrhenius plot of ln(*D_eff_*) versus 1/*T* for each variety. The activation energy values were 16.798 (*R*^2^ = 0.9982), 18.706 (*R*^2^ = 0.9611) and 22.999 (*R*^2^ = 0.9775) kJ/mol for Starkrimson, Idared and Janogold variety, respectively. These values fall within the typical range reported for food materials (12.7–110 kJ/mol) and are consistent with values previously reported for fruit pomaces and related materials [4,18,29,41]. The high *R*^2^ values (0.9611–0.9982) indicate excellent linear fit of the Arrhenius relationship, confirming the validity of the temperature dependence model.

The activation energy represents the minimum energy needed to initiate the diffusion of moisture and reflects the temperature sensitivity of the drying process. A lower activation energy suggests that the rate of moisture diffusion is less affected by changes in temperature, while higher values suggest greater temperature dependence. The relatively low activation energy values obtained in this study suggest that apple pomace drying is moderately temperature-sensitive, with moisture migration facilitated by the porous structure and high initial moisture content [2].

Comparison with literature reveals that the activation energy values determined in this study are lower than those reported by Kara and Doymaz (29.65 kJ/mol) for apple pomace [2], but comparable to values reported by Araujo et al. [4] (23.9 kJ/mol) for apple–ginger mixed pomace, and Wang et al. [5] (24.512 kJ/mol) for apple pomace. Other fruit pomace studies have reported activation energies ranging from 13 to 40 kJ/mol, depending on material properties and drying conditions [14,18]. The variation in activation energy among varieties could be attributed to differences in cellular structure, chemical composition, and moisture binding characteristics.

The inverse correlation between diffusivity and temperature observed in this study resulted in negative slopes in the Arrhenius plots, yielding positive activation energy values. However, this apparent contradiction suggests that the simplified Fickian diffusion model may not fully capture the complex drying behavior of apple pomace, particularly the effects of shrinkage and structural changes at different temperatures. More sophisticated models incorporating shrinkage, variable diffusivity, and multi-phase moisture transport may be required for complete mechanistic understanding [14,15,29,41].

### 3.5. Mathematical Modeling and Model Comparison

The experimental moisture ratio data were modeled using the seven mathematical models listed in Table 1 for all apple varieties and temperatures. Non-linear regression was employed to estimate the model parameters and the goodness of fit was evaluated using *R*^2^, *RMSE*, *χ*^2^, and *MBE*. The statistical performance indicators for selected models are shown in Table 3.

Only four representative models are shown for clarity. Complete results for all seven models showed similar ranking patterns.

Among all tested models, the Midilli–Kucuk model consistently performed better across all varieties and temperatures, with *R*^2^ values exceeding 0.999, *RMSE* values below 0.01, *χ*^2^ values below 0.0001, and *MBE* values near zero [2,4]. This model’s excellent fit was attributed to its flexible four-parameter structure combining an exponential term with power-law exponent and a linear term, allowing it to reflect both the early phase of moisture removal and the gradual approach to equilibrium [19,31,34].

The Page model also showed excellent predictive capability, ranking second with *R*^2^ values above 0.997, *RMSE* values of 0.016–0.019, and low *χ*^2^ values. The Page model’s two-parameter exponential form with power-law exponent provides good flexibility while maintaining simplicity, making it attractive for practical applications [11,32].

The Logarithmic model performed well, with *R*^2^ values above 0.996 and comparable *RMSE* and *χ*^2^ values [19,31].

The Newton (Lewis) model, despite its simplicity (single parameter), showed acceptable performance with *R*^2^ values above 0.98, but consistently ranked lower than the more complex models [33]. The Newton model’s single exponential form cannot capture the non-linear drying behavior as effectively as the models with additional parameters [8].

Other models tested (Modified Page, Henderson–Pabis, Modified Henderson–Pabis) showed intermediate performance, with *R*^2^ values varying between 0.985 and 0.998, as a function of variety and temperature [5,31].

The statistical indicators showed consistent patterns across varieties and temperatures, with minimal variation in model ranking. This consistency suggested that the selected models were robust and applicable across the range of conditions studied [2,19]. The near-zero *MBE* values for the best-fit models indicated the absence of systematic bias in predictions, confirming model reliability [33,42].

The superior performance of the Midilli–Kucuk model in this study was consistent with numerous reports in the literature for apple pomace and related fruit materials [2,34,43,44]. Kara and Doymaz [2] reported that the Midilli model best described apple pomace drying at 50–70 °C. Similarly, studies on apple slices, apricot pomace, and pomegranate arils have identified the Midilli model as providing the most accurate fit among multiple models tested.

The Page model’s strong performance is also well-documented in fruit drying literature, often ranking among the top two or three models. Its simplicity relative to the Midilli–Kucuk model makes it attractive for engineering applications where model complexity must be balanced against predictive accuracy.

The Midilli–Kucuk model demonstrated superior statistical performance. This can be attributed to its greater structural flexibility, as it includes four parameters (‘k’, ‘n’, ‘a’, and ‘b’), compared with the two parameters of the simpler Page model, which nevertheless also exhibited good predictive capability. From a practical perspective, the additional parameters of the Midilli–Kucuk model allow it to capture subtle deviations in drying behavior during both the initial and final stages of the drying process. These stages are particularly important for process control and endpoint determination in industrial applications. The parameter ‘b’ accounts for the influence of external drying conditions, whereas ‘a’ and ‘n’ describe the internal moisture transport characteristics of the material. Consequently, although the model involves greater computational complexity, its improved predictive accuracy is technologically significant for industrial implementation, where precise control of the final moisture content is essential for product stability and shelf life.

### 3.6. Color

The values of the CIEL*a*b* color parameters of apple pomace powders are presented in Table 4. For all varieties, the powders exhibited higher L* (lightness) values as the drying temperature increased. The observed increase in powder lightness can be attributed to the reduction in drying time at higher temperatures, thereby decreasing the incidence of enzymatic and non-enzymatic browning. No significant impact of drying temperature was observed on the a* and b* values. However, significant variations were observed among the varieties (*p* < 0.05), with the highest a* and b* values recorded for Jonagold, followed by Idared and Starkrimson, this order also being maintained for the Chrome (C*) values. In terms of lightness, Starkrimson exhibited the highest L* values, followed by Idared and Jonagold.

### 3.7. Water Activity (a_w_) and Rehydration Rate

Water activity progressively decreased in all samples with increasing drying temperature (Table 5). In many cases, the reduction in water activity was statistically significant (*p* < 0.05).

The rehydration behavior of dried foods serves as a key quality indicator, revealing the level of structural and cellular damage caused during processing. Rehydration ratio is affected by the dehydration method and its parameters, the product composition and microstructure, the predrying treatments and rehydration conditions [45].

A progressive decrease in the rehydration ratio was observed with increasing dehydration temperature in apple pomace powders from all apple varieties. Significant differences were also recorded between varieties. Higher values of the rehydration ratio were recorded in Starkrimson followed by Jonagold and Idared, for the same dehydration temperature. Previous studies have similarly shown that increasing the dehydration temperature reduces the rehydration ratio of fruit and vegetable powders [11,46,47].

The decrease in rehydration ratio at higher drying temperatures can be attributed to increased shrinkage and deterioration of the material’s open structure and porosity [48], which worsened the rehydration characteristics. According to Mahiuddin et al. [49], material shrinkage during drying is primarily caused by two factors: (a) the inability of tissues to maintain their structure when water vacates various spaces that are occupied by air, and (b) the collapse of the structure.

### 3.8. Proximate Composition

The results on the proximal composition of apple pomace powders are presented in Table 6. The apple pomace powders obtained by drying at higher temperatures (68 °C) showed slightly higher values of dry matter content, results that are consistent with the slightly lower values of the water activity of these samples.

The protein content of apple pomace powders was not significantly influenced (*p* > 0.05) by the dehydration temperature, nor were the cellulose and mineral content. The fat content tended to increase with increasing drying temperature in Starkrimson and Idared pomace powders, while an inverse trend was found in Jonagold pomace powder.

Regarding the differences between varieties, a slightly but significantly (*p* < 0.05) lower content of protein, fat, cellulose and mineral substances was found in the Jonagold samples compared to Starkrimson and Idared, but also a lower dry matter content of powders obtained from this variety.

The ash content was not significantly affected by dehydration temperature; however, significant differences were observed among varieties. Phosphorus (0.07–0.08%), calcium (0.06–0.10%), magnesium (0.02–0.36%), and iron (31.8–38.3 mg/kg d.w.) have been reported as the predominant minerals in apple pomace [50].

In a comprehensive review, by averaging data provided in earlier studies, Waldbauer et al. [51] reported the proximate composition of dried apple pomace as 92.67% moisture, 36.89% total fiber, 3.73% protein, and 1.88% ash.

The results obtained for the proximate composition of apple pomace powders in the present study align with previously published data [52,53,54,55]. Those studies reported a dry matter content ranging from 89.5 to 95.6%, protein levels between 1.2 and 4.7%, fat content from 0.6 to 4.2%, and ash content between 1.5 and 2.5%.

### 3.9. Titratable Acidity, Total Phenolic Content and DPPH Radical Scavenging Activity

The drying temperature did not have a significant effect (*p* > 0.05) on the titratable acidity of apple pomace powders (Table 7); however, significant differences were observed among varieties. The highest titratable acidity was found in Idared samples, followed by Starkrimson and Jonagold.

No significant differences were recorded in the phenolic content of samples dried at 57 and 63 °C while increasing the drying temperature from 63 to 68 °C caused a significant decrease in the total phenolic content only in Starkrimson pomace powder. Many studies have been carried out to determine how different drying methods influence the recovery and modification of phenolic compounds [56]. Some previous studies have shown that drying may lead to the degradation of certain heat-sensitive phenolic compounds [57]. However, in a review investigating the impact of drying temperature on total phenolic and total flavonoid contents in fruits and vegetables, Červenka et al. [58] reported that these compounds were generally not markedly affected by drying temperature. An exception was observed for total phenolic content in plant-based foods dried at 70–80 °C, where a significant reduction was noted compared with samples dried at 40 °C.

Raising the air temperature significantly accelerated the drying kinetics, shortening the total drying time from 210 min at 57 °C to 180 min at 63–68 °C for the Starkrimson variety, and from 180 min at 57 °C to 150 min at 63–68 °C for the Idared and Jonagold varieties. A shorter process time helps to preserve phenolic compounds by shortening the exposure time to oxygen and by lowering enzymatic browning despite the higher temperature [56]. Samoticha et al. [59] found that the longer drying time of chokeberry fruit at 50 °C in convective drying resulted in higher degradation of polyphenolic compounds than in short drying at 70 °C. Within the temperature range of 57–68 °C, commonly applied in drying plant-based materials, the findings suggest that 63 °C provides the most favorable balance between efficient moisture removal and the preservation of phenolic compounds in apple pomace.

The total phenolic content of apple pomace powders ranged from 166.55 to 214.50 mg/100 g. Previously, Pollini et al. [60] reported total phenolic content of 80–106 g/100 g in fresh apple pomace while other studies reported values between 47 and 1415 mg GAE/100 g in dried apple pomace [51]. A review of the literature by Antonic et al. [61] reported total phenolic content ranging from 170 to 990 mg/100 g in dried apple pomace.

RSA was not significantly affected (*p* > 0.05) by the dehydration temperature (Table 7), although significant differences were observed among varieties. The highest total phenolic content was found in pomace powders from the Starkrimson variety, followed by Jonagold and Idared. Powders from the Idared variety also exhibited the lowest RSA, whereas no significant differences (*p* > 0.05) were found between the antioxidant activities of Starkrimson and Jonagold pomace powders.

### 3.10. Phenolic Compounds

The contents of individual phenolic compounds in apple pomace powders, quantified by RP-HPLC, are presented in Table 8. Previous research has consistently shown that apple pomace possesses a high content of natural antioxidants, including quercetin glycosides, phloretin glycosides, chlorogenic acid and other phenolic compounds exhibiting strong antioxidant activity [50].

The most abundant quantified phenolic compounds were rutin (10.94–37.26 mg/100 g) and chlorogenic acid (10.89–17.17 mg/100 g), followed by epicatechin and vanillic acid. In agreement with these results, previous studies reported 2.6–229.8 mg chlorogenic acid/100 g, 0.42–64 mg epicatechin/100 g, 0.1–12.7 mg catechin/100 g, and 0.3–28 mg caffeic acid/100 g in dehydrated apple pomace [61]. Consistent with these findings, Gaharwar et al. [62] confirmed the presence of multiple polyphenols in apple pomace extracts, including rutin, syringic acid, chlorogenic acid, *p*-coumaric acid, catechin, epicatechin, and ferulic acid. Despite having the lowest total phenolic content, Idared powders contained the highest rutin levels. Starkrimson powders were richest in chlorogenic acid, while Jonagold powders were notable for their elevated vanillic and trans-cinnamic acid contents, alongside the lowest levels of catechin and caffeic acid.

Phenolic compounds are a class of biologically active plant secondary metabolites with multiple functional properties determined by their unique chemical structure and properties, comprising simple phenolics, coumarins, lignans, flavonoids, isoflavonoids, anthocyanins, proanthocyanidins and stilbenes. They exhibit a wide range of beneficial biological activities, including antioxidant, anti-inflammatory, antitumor, and cardioprotective effects. The intake of phenolic compounds may contribute to the prevention of various chronic conditions, particularly those associated with oxidative stress, as well as certain cardiovascular and neurodegenerative diseases. Various drying techniques and processing conditions significantly influence the level of phenolic compounds in dried products. As a result of the difference in their molecular structures, the concentrations of individual phenolic compounds are typically influenced by temperature to varying extents. Therefore, optimal drying temperatures may differ not only among specific phenolics within the same product but also across different products. Previous studies revealed that, in convective drying, the ideal drying temperatures for retaining polyphenols, flavonoids, and glycosides were 55–60 °C, 60–70 °C, and 45–50 °C, respectively [63].

A general decreasing trend in the content of phenolic compounds was noted in the present study with increasing dehydration temperature; however, these differences were not always statistically significant (*p* > 0.05). Rutin and epicatechin were the flavonoids most strongly affected by increasing the temperature to 68 °C. Among the phenolic acids, chlorogenic acid was the most sensitive to temperature elevation, showing noticeable losses even at 63 °C. Changes in phenolic compounds during drying can be influenced by several factors, including their degradation, especially during extended drying at higher temperatures, the release of bound phenolics, the liberation of phenolic acid derivatives, and other related processes [56]. The molecular structure of phenols, flavonoids, and glycosides contains a benzene ring and a variable number of hydroxyl groups (–OH), which exhibit strong reducing properties. When heated, the hydroxyl groups are often oxidized to form aldehyde, ketone, or carboxyl groups. The rate of degradation is primarily influenced by temperature, oxygen levels, and moisture content [63].

### 3.11. Organic Acids

Organic acids act as key flavor components that significantly influence fruit quality and sensory characteristics. Together with soluble sugars, they shape the distinctive taste and aroma of fruit products, with their specific types and relative proportions playing a crucial role in determining consumer acceptance [64]. As expected, the organic acid profile of the powders was dominated by malic acid, followed by tartaric and oxalic acids, while only minor amounts of citric acid and ascorbic acid were quantified (Table 9). Malic acid content tended to decrease with increasing dehydration temperature, with a significant reduction noted at 68 °C. Tartaric and ascorbic acid levels were not significantly affected by dehydration temperature (*p* > 0.05), whereas oxalic acid content decreased significantly (*p* < 0.05). Therefore, raising the air-drying temperature from 63 to 68 °C would markedly modify the taste and aroma of apple pomace powder. This change is undesirable when considering its use as an ingredient or as a substitute for apple powder in food products.

The organic acid profile was significantly affected by apple variety. Pomace powders from the Idared variety exhibited the highest malic acid content, corresponding to the highest titratable acidity. In contrast, Jonagold powders contained the highest levels of tartaric acid, while Starkrimson powders were notable for the highest citric and ascorbic acid contents. These findings align with earlier studies: Queji et al. [65] reported malic acid concentrations ranging from 0.905 to 0.19% (*w*/*w*) in the dried pomace of 26 apple varieties, while Sato et al. [66] reported malic acid levels between 0.73 and 1.32 g/100 g in pomace from 11 apple varieties.

## 4. Conclusions

This study focused on the thin-layer drying kinetics of apple pomace from three commercial varieties (Starkrimson, Idared, and Jonagold) under forced convection at 57 °C, 63 °C, and 68 °C. Seven mathematical models were evaluated using comprehensive statistical indicators to select the model that best describes the drying behavior. Across all varieties, the drying process exhibited a characteristic falling-rate regime, with no constant-rate period observed, which indicated that internal mass transfer—primarily effective moisture diffusion—governed the moisture removal mechanism. Increasing the air temperature clearly accelerated the drying kinetics and reduced the overall drying time, reflecting enhanced moisture mobility and a higher driving force for heat and mass transfer. Water activity and rehydration ratio progressively decreased in apple pomace powders with increasing dehydration temperature. The powders exhibited a clear varietal trend in total phenolic content and antioxidant activity, ranked as Starkrimson > Jonagold > Idared. The results indicated that a drying temperature of 63 °C provides the most favorable balance between efficient moisture removal and the preservation of phenolic compounds in apple pomace. This approach supports the sustainable valorization of fruit by-products, converting waste from the fruit juice industry into valuable resources for new food applications within a circular economy framework.

## Figures and Tables

**Table 1:** Mathematical models applied to describe thin-layer drying kinetics of apple pomace.

Model Name	Model Equation	Reference
Lewis (Newton)	*MR* = exp(−*kt*)	[33]
Page	*MR* = exp(−*kt^n^*)	[11]
Modified Page	*MR* = exp(−(*kt*)*^n^*)	[24]
Henderson–Pabis	*MR* = *a* exp(−*kt*)	[5]
Logarithmic	*MR* = *a* exp(−*kt*) + *c*	[31]
Midilli–Kucuk	*MR* = *a* exp(−*kt^n^*) + *bt*	[34]
Modified Henderson–Pabis	*MR* = *a* exp(−*kt*) + *b* exp(−*gt*) + *c* exp(−*ht*)	[28]

**Table 2:** Effective moisture diffusivity and coefficient of determination for apple pomace varieties at different drying temperatures.

Apple Variety	Temperature (°C)	*R* ^2^	*D_eff_* × 10^−8^ (m^2^/s)
Starkrimson	57	0.9357	1.167
	63	0.9186	1.293
	68	0.9442	1.423
Idared	57	0.9501	1.321
	63	0.9379	1.548
	68	0.9556	1.641
Jonagold	57	0.9347	1.293
	63	0.9398	1.451
	68	0.9530	1.698

**Table 3:** Statistical performance indicators of selected mathematical models for apple pomace drying.

Variety	Temperature (°C)	Model	*R* ^2^	*RMSE*	*χ* ^2^	*MBE*
Starkrimson	57	Newton	0.9857	0.0421	0.00177	0.0012
		Page	0.9983	0.0165	0.00027	−0.0008
		Midilli–Kucuk	0.9989	0.0078	0.00006	0.0002
		Logarithmic	0.9968	0.0199	0.00040	0.0005
	63	Newton	0.9823	0.0468	0.00219	0.0015
		Page	0.9971	0.0189	0.00036	−0.0010
		Midilli–Kucuk	0.9993	0.0092	0.00008	0.0003
		Logarithmic	0.9961	0.0220	0.00048	0.0007
	68	Newton	0.9845	0.0438	0.00192	0.0013
		Page	0.9975	0.0176	0.00031	−0.0009
		Midilli–Kucuk	0.9994	0.0086	0.00007	0.0002
		Logarithmic	0.9965	0.0208	0.00043	0.0006
Idared	57	Newton	0.9841	0.0444	0.00197	0.0014
		Page	0.9974	0.0179	0.00032	−0.0009
		Midilli–Kucuk	0.9994	0.0086	0.00007	0.0002
		Logarithmic	0.9964	0.0211	0.00045	0.0006
	63	Newton	0.9829	0.0460	0.00212	0.0015
		Page	0.9972	0.0186	0.00035	−0.0010
		Midilli–Kucuk	0.9993	0.0093	0.00009	0.0003
		Logarithmic	0.9962	0.0217	0.00047	0.0007
	68	Newton	0.9852	0.0428	0.00183	0.0013
		Page	0.9976	0.0172	0.00030	−0.0009
		Midilli–Kucuk	0.9995	0.0079	0.00006	0.0002
		Logarithmic	0.9967	0.0202	0.00041	0.0006
Jonagold	57	Newton	0.9835	0.0452	0.00204	0.0014
		Page	0.9973	0.0183	0.00033	−0.0009
		Midilli–Kucuk	0.9994	0.0087	0.00008	0.0002
		Logarithmic	0.9963	0.0214	0.00046	0.0006
	63	Newton	0.9826	0.0464	0.00215	0.0015
		Page	0.9971	0.0189	0.00036	−0.0010
		Midilli–Kucuk	0.9993	0.0094	0.00009	0.0003
		Logarithmic	0.9961	0.0220	0.00048	0.0007
	68	Newton	0.9848	0.0434	0.00188	0.0013
		Page	0.9975	0.0176	0.00031	−0.0009
		Midilli–Kucuk	0.9994	0.0085	0.00007	0.0002
		Logarithmic	0.9966	0.0205	0.00042	0.0006

**Table 4:** Effects of drying temperature and variety on CIEL*a*b* color parameters of apple pomace powders.

Variety	Temperature (°C)	L*	a*	b*	C*	h*
Starkrimson	57	55.08 ± 0.40 ^bA^	15.94 ± 0.22 ^aC^	29.65 ± 0.57 ^aC^	33.66 ± 0.60 ^aC^	61.74 ± 0.21 ^bA^
	63	54.64 ± 0.65 ^bA^	16.04 ± 0.47 ^aC^	29.24 ± 1.00 ^aC^	32.71 ± 0.45 ^bC^	61.11 ± 0.13 ^cA^
	68	58.56 ± 0.52 ^aA^	15.65 ± 0.38 ^aC^	29.49 ± 0.73 ^aC^	33.38 ± 0.81 ^abC^	62.06 ± 0.21 ^aA^
Idared	57	50.54 ± 0.42 ^cB^	17.08 ± 0.59 ^aB^	30.95 ± 0.77 ^aB^	35.35 ± 0.95 ^aB^	61.12 ± 0.35 ^aA^
	63	52.24 ± 0.22 ^aB^	17.37 ± 0.22 ^aB^	31.35 ± 0.36 ^aB^	35.84 ± 0.42 ^aB^	61.00 ± 0.14 ^aA^
	68	51.43 ± 0.77 ^bB^	17.17 ± 0.16 ^aB^	31.00 ± 0.25 ^aB^	35.44 ± 0.28 ^aB^	61.01 ± 0.14 ^aB^
Jonagold	57	44.34 ± 0.88 ^bC^	20.76 ± 0.70 ^aA^	34.21 ± 0.73 ^aA^	40.02 ± 0.82 ^aA^	58.74 ± 0.84 ^bB^
	63	45.85 ± 1.49 ^abC^	20.22 ± 0.86 ^aA^	34.63 ± 1.14 ^aA^	40.11 ± 1.19 ^aA^	59.72 ± 1.14 ^abB^
	68	47.06 ± 0.96 ^aC^	19.74 ± 0.82 ^aA^	35.10 ± 0.86 ^aA^	40.27 ± 1.08 ^aA^	60.66 ± 0.69 ^aB^

Different lowercase letters in each column indicate significant differences between samples from the same variety dried at different temperatures (*p* < 0.05) and different uppercase letters in each column indicate significant differences between samples from different varieties (*p* < 0.05) dried at the same temperature.

**Table 5:** Effects of drying temperature and variety on the rehydration ratio and water activity (a_w_) of apple pomace powders.

Variety	Temperature (°C)	Rehydration Ratio	Water Activity (a_w_)
Starkrimson	57	5.89 ± 0.15 ^aA^	0.29 ± 0.02 ^aA^
	63	5.82 ± 0.21 ^aA^	0.27 ± 0.02 ^abB^
	68	5.29 ± 0.18 ^bA^	0.25 ± 0.01 ^bB^
Idared	57	5.18 ± 0.25 ^aAB^	0.31 ± 0.02 ^aA^
	63	5.00 ± 0.09 ^aB^	0.29 ± 0.01 ^aAB^
	68	4.88 ± 0.16 ^aB^	0.24 ± 0.01 ^bB^
Jonagold	57	5.63 ± 0.17 ^aB^	0.32 ± 0.03 ^aA^
	63	5.34 ± 0.15 ^aB^	0.31 ± 0.02 ^aA^
	68	5.33 ± 0.13 ^aA^	0.29 ± 0.02 ^aA^

Different lowercase letters in each column indicate significant differences between samples from the same variety dried at different temperatures (*p* < 0.05) and different uppercase letters in each column indicate significant differences between samples from different varieties (*p* < 0.05) dried at the same temperature.

**Table 6:** Effects of drying temperature and variety on the dry matter, protein, fat, cellulose and mineral content of apple pomace powders.

Variety	Temperature (°C)	Dry Matter (%)	Protein (%)	Fat (%)	Cellulose (%)	Ash (%)
Starkrimson	57	93.38 ± 0.67 ^aA^	3.49 ± 0.18 ^aA^	1.44 ± 0.08 ^bA^	8.94 ± 0.37 ^aB^	1.28 ± 0.06 ^aA^
	63	93.66 ± 0.71 ^aA^	3.37 ± 0.15 ^aA^	1.58 ± 0.09 ^bA^	9.21 ± 0.33 ^aB^	1.20 ± 0.05 ^aA^
	68	94.34 ± 0.59 ^aA^	3.24 ± 0.20 ^aA^	1.75 ± 0.08 ^aA^	9.07 ± 0.28 ^aAB^	1.22 ± 0.06 ^aA^
Idared	57	91.75 ± 0.55 ^bB^	2.83 ± 0.12 ^bC^	1.56 ± 0.09 ^bA^	10.02 ± 0.43 ^aA^	1.22 ± 0.05 ^aA^
	63	93.61 ± 0.52 ^aA^	3.17 ± 0.18 ^aA^	1.62 ± 0.09 ^abA^	9.64 ± 0.36 ^bA^	1.16 ± 0.04 ^aA^
	68	94.03 ± 0.89 ^aA^	3.14 ± 0.21 ^aA^	1.78 ± 0.09 ^aA^	9.38 ± 0.24 ^bA^	1.18 ± 0.05 ^aA^
Jonagold	57	91.12 ± 0.25 ^bB^	3.14 ± 0.11 ^aB^	1.26 ± 0.06 ^aB^	8.46 ± 0.29 ^aC^	0.93 ± 0.04 ^bB^
	63	91.33 ± 0.47 ^bB^	3.21 ± 0.14 ^aA^	1.15 ± 0.04 ^bB^	8.61 ± 0.33 ^aC^	0.98 ± 0.03 ^bB^
	68	92.49 ± 0.61 ^aB^	3.03 ± 0.09 ^aA^	0.93 ± 0.04 ^cB^	8.74 ± 0.35 ^aB^	1.08 ± 0.05 ^aB^

Different lowercase letters in each column indicate significant differences between samples from the same variety dried at different temperatures (*p* < 0.05) and different uppercase letters in each column indicate significant differences between samples from different varieties (*p* < 0.05) dried at the same temperature.

**Table 7:** Effects of drying temperature and variety on titratable acidity, total phenolic content and DPPH radical scavenging activity of apple pomace powders.

Variety	Temperature (°C)	Titratable Acidity (g Malic Acid/100 g)	Total Phenolic Content (mg GAE/100 g)	DPPH Radical Scavenging Activity (mmol Trolox/100 g)
Starkrimson	57	1.47 ± 0.09 ^aB^	214.05 ± 8.98 ^aA^	0.65 ± 0.06 ^aA^
	63	1.36 ± 0.08 ^aB^	214.50 ± 9.66 ^aA^	0.74 ± 0.04 ^aA^
	68	1.47 ± 0.06 ^aB^	196.32 ± 8.57 ^bA^	0.69 ± 0.00 ^aA^
Idared	57	2.14 ± 0.11 ^aA^	169.05 ± 9.91 ^aB^	0.56 ± 0.02 ^aA^
	63	2.14 ± 0.07 ^aA^	166.55 ± 5.50 ^aC^	0.58 ± 0.02 ^aB^
	68	2.01 ± 0.09 ^aA^	177.45 ± 8.72 ^aB^	0.58 ± 0.01 ^aB^
Jonagold	57	1.21 ± 0.08 ^aC^	182.91 ± 7.96 ^bB^	0.65 ± 0.06 ^aA^
	63	1.21 ± 0.06 ^aC^	194.50 ± 6.82 ^aB^	0.65 ± 0.06 ^aA^
	68	1.25 ± 0.09 ^aC^	198.82 ± 6.86 ^aA^	0.68 ± 0.03 ^aA^

Different lowercase letters in each column indicate significant differences between samples from the same variety dried at different temperatures (*p* < 0.05) and different uppercase letters in each column indicate significant differences between samples from different varieties (*p* < 0.05) dried at the same temperature.

**Table 8:** Effects of drying temperature and variety on the phenolic content (mg/100 g) of apple pomace powders.

Variety	Temperature (°C)	Vanillic Acid	Rutin	Quercetin	Galic Acid	Catechin Hydrate	Syringic Acid
Starkrimson	57	0.79 ± 0.01 ^bC^	14.90 ± 0.31 ^aC^	0.07 ± 0.01 ^aB^	0.03 ± 0.00 ^aA^	0.52 ± 0.01 ^aB^	nd
	63	0.88 ± 0.03 ^aC^	12.59 ± 0.20 ^bC^	0.07 ± 0.01 ^aB^	0.02 ± 0.00 ^aA^	0.45 ± 0.01 ^bB^	nd
	68	0.88 ± 0.05 ^aC^	10.94 ± 0.61 ^cB^	0.08 ± 0.01 ^aB^	0.01 ± 0.00 ^aA^	0.41 ± 0.01 ^cB^	nd
Idared	57	1.11 ± 0.03 ^bB^	37.26 ± 0.68 ^aA^	0.09 ± 0.00 ^aA^	0.02 ± 0.00 ^aA^	0.65 ± 0.05 ^aA^	nd
	63	1.18 ± 0.01 ^abB^	24.25 ± 1.22 ^bB^	0.09 ± 0.01 ^aA^	0.02 ± 0.00 ^aA^	0.65 ± 0.01 ^aA^	nd
	68	1.35 ± 0.07 ^aB^	22.98 ± 0.18 ^cA^	0.12 ± 0.02 ^aA^	0.01 ± 0.00 ^aA^	0.48 ± 0.01 ^bA^	nd
Jonagold	57	1.52 ± 0.06 ^aA^	32.57 ± 0.89 ^aB^	0.02 ± 0.00 ^aC^	0.02 ± 0.00 ^aA^	0.25 ± 0.01 ^aC^	nd
	63	1.37 ± 0.11 ^aA^	32.87 ± 0.23 ^aA^	0.03 ± 0.01 ^aC^	0.02 ± 0.00 ^aA^	0.26 ± 0.02 ^aC^	nd
	68	1.47 ± 0.05 ^aA^	23.45 ± 0.93 ^bA^	0.02 ± 0.01 ^aC^	0.01 ± 0.00 ^aA^	0.26 ± 0.01 ^aC^	nd
		**Epicatechin**	***Trans*** **-** **cinnamic acid**	**Chlorogenic acid**	**Caffeic acid**	***p*** **-Coumaric acid**	**Ferulic acid**
Starkrimson	57 °C	5.30 ± 0.14 ^aA^	0.24 ± 0.01 ^aA^	17.17 ± 0.56 ^aA^	0.42 ± 0.02 ^aA^	0.02 ± 0.00 ^aB^	0.05 ± 0.01 ^aA^
	63 °C	5.18 ± 0.26 ^aA^	0.14 ± 0.02 ^bB^	13.48 ± 0.27 ^bA^	0.43 ± 0.01 ^aA^	0.03 ± 0.00 ^aA^	0.06 ± 0.01 ^aA^
	68 °C	4.63 ± 0.21 ^aA^	0.09 ± 0.02 ^cB^	13.51 ± 0.59 ^bA^	0.42 ± 0.01 ^aA^	0.05 ± 0.02 ^aA^	0.07 ± 0.01 ^aA^
Idared	57 °C	4.27 ± 0.15 ^aB^	0.13 ± 0.03 ^aB^	11.05 ± 0.55 ^aB^	0.37 ± 0.02 ^aB^	0.04 ± 0.00 ^aA^	0.05 ± 0.00 ^aA^
	63 °C	2.26 ± 0.22 ^bB^	0.12 ± 0.03 ^aB^	11.03 ± 0.34 ^aB^	0.36 ± 0.01 ^aB^	0.03 ± 0.01 ^aA^	0.05 ± 0.00 ^aA^
	68 °C	1.27 ± 0.15 ^cB^	0.13 ± 0.01 ^aB^	10.89 ± 0.29 ^aB^	0.35 ± 0.01 ^aB^	0.04 ± 0.00 ^aA^	0.06 ± 0.00 ^aA^
Jonagold	57 °C	5.04 ± 0.21 ^aA^	0.24 ± 0.02 ^aA^	11.83 ± 0.40 ^aB^	0.19 ± 0.01 ^aC^	0.03 ± 0.00 ^aAB^	0.05 ± 0.00 ^aA^
	63 °C	4.98 ± 0.24 ^aA^	0.23 ± 0.01 ^aA^	11.40 ± 0.27 ^aB^	0.20 ± 0.03 ^aC^	0.04 ± 0.00 ^aA^	0.06 ± 0.01 ^aA^
	68 °C	4.99 ± 0.04 ^aA^	0.23 ± 0.01 ^aA^	11.69 ± 0.05 ^aB^	0.20 ± 0.01 ^aC^	0.03 ± 0.00 ^aA^	0.06 ± 0.00 ^aA^

Different lowercase letters in each column indicate significant differences between samples from the same variety dried at different temperatures (*p* < 0.05) and different uppercase letters in each column indicate significant differences between samples from different varieties (*p* < 0.05) dried at the same temperature.

**Table 9:** Effects of drying temperature and variety on the organic acid content (mg/100 g) of apple pomace powders.

Variety	Temperature (°C)	Malic Acid	Tartaric Acid	Citric Acid	Oxalic Acid	Ascorbic Acid
Starkrimson	57	1276.91 ± 57.70 ^aB^	191.32 ± 8.66 ^aB^	6.72 ± 0.13 ^aA^	32.04 ± 0.98 ^a^	0.75 ± 0.04 ^aA^
	63	1148.78 ± 48.84 ^bB^	187.90 ± 7.75 ^aB^	6.55 ± 0.16 ^bA^	28.91 ± 0.67 ^b^	0.78 ± 0.03 ^aA^
	68	1202.84 ± 55.66 ^abB^	195.05 ± 6.88 ^aB^	6.10 ± 0.26 ^cA^	28.07 ± 0.71 ^b^	0.76 ± 0.05 ^aA^
Idared	57	1769.02 ± 75.54 ^aA^	185.31 ± 8.11 ^aB^	3.93 ± 0.12 ^aB^	25.09 ± 1.11 ^a^	0.54 ± 0.01 ^aB^
	63	1784.88 ± 66.79 ^aA^	189.75 ± 7.77 ^aB^	3.24 ± 0.15 ^bB^	20.74 ± 0.66 ^b^	0.53 ± 0.03 ^aB^
	68	1631.67 ± 49.95 ^bA^	190.15 ± 8.32 ^aB^	3.99 ± 0.18 ^aB^	18.15 ± 0.71 ^c^	0.49 ± 0.02 ^aB^
Jonagold	57	1196.07 ± 48.88 ^aB^	212.79 ± 9.66 ^aA^	2.28 ± 0.14 ^aC^	36.25 ± 1.45 ^a^	0.37 ± 0.02 ^aC^
	63	1146.52 ± 45.77 ^aB^	207.02 ± 8.76 ^aA^	2.13 ± 0.12 ^abC^	29.04 ± 1.28 ^b^	0.35 ± 0.02 ^aC^
	68	1067.22 ± 39.98 ^bC^	213.97 ± 10.13 ^aA^	2.01 ± 0.09 ^bC^	29.39 ± 1.36 ^b^	0.35 ±0.01 ^aC^

Different lowercase letters in each column indicate significant differences between samples from the same variety dried at different temperatures (*p* < 0.05) and different uppercase letters in each column indicate significant differences between samples from different varieties (*p* < 0.05) dried at the same temperature.

## Data Availability

The original contributions presented in the study are included in the article/Supplementary Materials. Further inquiries can be directed to the corresponding author.

## Supplementary Materials

The following supporting information can be downloaded at: https://www.mdpi.com/article/10.3390/foods15061090/s1, Figure S1. Appearance of the apple pomace uniformly spread in a thin layer (2 mm thickness before drying) on parchment paper covering a circular surface with 26 cm diameter (1) and of the apple pomace powder (2) for Starkrimson (a), Idared (b) and Jonagold (c) varieties.
