# Supplementary material for: Thin-Layer Drying Kinetics and Quality Attributes of Apple Pomace Powders from Different Varieties
Source: Foods. 2026 Mar 20;15(6):1090. doi: 10.3390/foods15061090 (PMC13025561; doi:10.3390/foods15061090)
Supplement: Supplementary file 1 [file foods-15-01090-s001.zip › foods-4173504-supplementary.pdf]

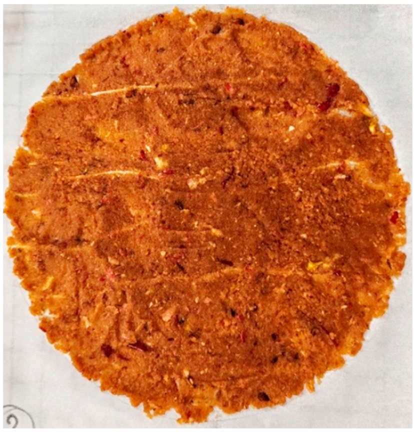

1a

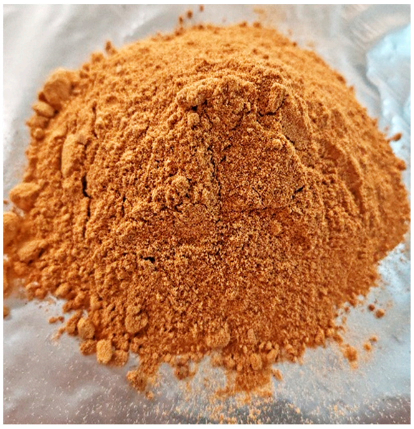

2a

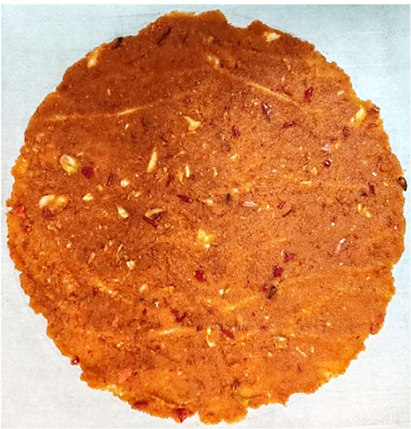

1b

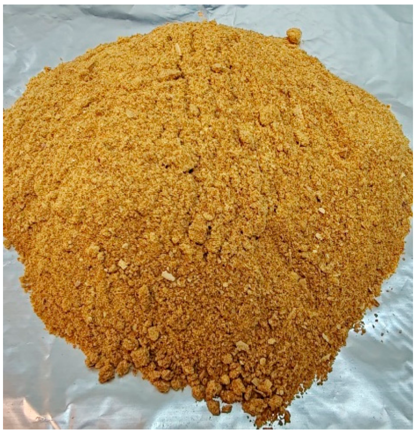

2b

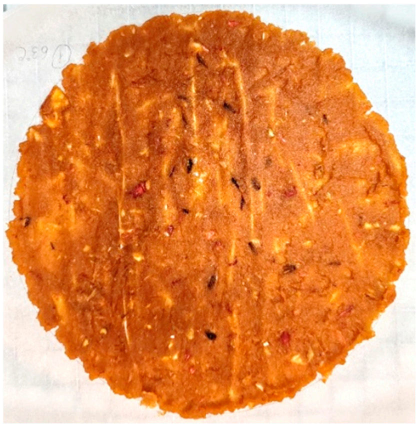

1c

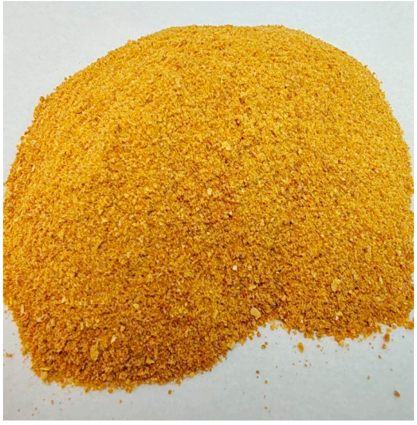

2c

Figure S1. Appearance of the apple pomace uniformly spread in a thin layer (2 mm thickness before drying) on parchment paper covering a circular surface with 26 cm diameter (1) and of the apple pomace powder (2) for Starkrimson (a), Idared (b) and Jonagold (c) varieties.
